# Genomic profiling of lung adenocarcinoma patients reveals therapeutic targets and confers clinical benefit when standard molecular testing is negative

**DOI:** 10.18632/oncotarget.8138

**Published:** 2016-03-16

**Authors:** Sun Min Lim, Eun Young Kim, Hye Ryun Kim, Siraj M. Ali, Joel R. Greenbowe, Hyo Sup Shim, Hyun Chang, Seungtaek Lim, Soonmyung Paik, Byoung Chul Cho

**Affiliations:** ^1^ Department of Internal Medicine, Division of Medical Oncology, CHA Bundang Hospital, CHA University, Korea; ^2^ Department of Internal Medicine, Division of Medical Oncology, Yonsei University College of Medicine, Seoul, Korea; ^3^ Department of Pulmonology, Yonsei University College of Medicine, Seoul, Korea; ^4^ Clinical Development, Foundation Medicine, Inc, Cambridge, MA, USA; ^5^ Department of Pathology, Yonsei University College of Medicine, Seoul, Korea; ^6^ Hematology and Medical Oncology, International St Mary's Hospital, Catholic Kwandong University, College of Medicine, Incheon, Korea; ^7^ Hematology and Medical Oncology, Wonju Severance Christianity Hospital, Wonju, Korea; ^8^ Severance Biomedical Science Institute, Yonsei University College of Medicine, Seoul, Korea

**Keywords:** lung adenocarcinoma, next-generation sequencing, cancer gene test, genomic profiling

## Abstract

**Background:** Identification of clinically relevant oncogenic drivers in advanced cancer is critical in selecting appropriate targeted therapy. Using next-generation sequencing (NGS)-based clinical cancer gene assay, we performed comprehensive genomic profiling (CGP) of advanced cases of lung adenocarcinoma.

**Methods:** Formalin-fixed paraffin-embedded tumors from 51 lung adenocarcinoma patients whose tumors previously tested negative for *EGFR/KRAS/ALK* by conventional methods were collected, and CGP was performed via hybridization capture of 4,557 exons from 287 cancer-related genes and 47 introns from 19 genes frequently rearranged in cancer.

**Results:** Genomic profiles of all 51 cases were obtained, with a median coverage of 564x and a total of 190 individual genomic alterations (GAs). GAs per specimen was a mean of 3.7 (range 0-10).Cancer genomes are characterized by 50% (80/190) non-synonymous base substitutions, 15% (29/190) insertions or deletion, and 3% (5/190) splice site mutation. *TP53* mutation was the most common GAs (15%, n=29/190), followed by *CDKN2A* homozygous loss (5%, n=10/190), *KRAS* mutation (4%, n=8/190), *EGFR* mutation (4%, n=8/190) and *MDM2* amplification (2%, n=5/190). As per NCCN guidelines, targetable GAs were identified in 16 patients (31%) (*BRAF* mutation [n=1], *EGFR* mutation [n=8], *ERBB2* mutation [n=4], *MET* amplification [n=1], *KIF5B-RET* rearrangement [n=2], *CCDC6-RET* rearrangement [n=1], *CD74-ROS1* rearrangement [n=1], *EZR-ROS1* rearrangement [n=5], and *SLC34A2-ROS1* rearrangement [n=1]).

**Conclusion:** Fifty eight percent of patients wild type by standard testing for *EGFR/KRAS/ALK* have GAs identifiable by CGP that suggest benefit from target therapy. CGP used when standard molecular testing for NSCLC is negative can reveal additional avenues of benefit from targeted therapy.

## BACKGROUND

The treatment of non-small-cell lung cancer has been revolutionized due to development of molecularly targeted therapy in genomically defined subsets of patients. The discovery of epidermal growth factor receptor (*EGFR*) mutation [1] and anaplastic lymphoma kinase (*ALK*) gene rearrangement are classic examples of oncogenic genomic alterations (GAs) that confer sensitivity to matched targeted therapy [2]. Patients with *EGFR* or *ALK* gene alterations have experienced significant survival benefit from targeted therapy as compared to conventional chemotherapy [3, 4]. Undoubtedly, tyrosine kinase inhibitors (TKI) for *EGFR* and *ALK* are currently the standard of care for *EGFR*-mutant and *ALK*-rearranged lung cancer patients, respectively.

However, most lung adenocarcinomas lack an identifiable driver oncogene such as the above and are therefore still treated with conventional chemotherapy. Therefore, maximally identifying actionable GAs in advanced lung cancer patients is essential to improve clinical outcome. The use of comprehensive genomic profiling (CGP) enabled identification of oncogenic alterations that would previously been missed by conventional testing, as this provides simultaneous detection of alterations in multiple cancer genes with higher sensitivity and specificity. Collaborated efforts using NGS has increased the diversity and expanded the range of possible targetable alterations in lung adenocarcinomas. A recent study by the Cancer Genome Atlas (TCGA) revealed potential novel drivers such as *NF1, MET, ERBB2* and *RIT1* which occur in 13% of cases, and enriched in samples lacking an activated oncogene [5]. These alterations, which occurred in previously oncogene-negative subsets, may be single-gene driver events similar to *EGFR* and *ALK*.

Recently, rather than sequencing the entire genome or exome, CGP which include genes that show frequent alterations in cancer were developed to save the amount of tissue, time and effort to perform sequencing. These panels use PCR capture-based NGS assay that allow deep targeted sequencing of genes of interest from limited formalin-fixed, paraffin-embedded (FFPE) specimens [6]. Since incorporating NGS into routine oncologic practice requires accurate genomic profiling in a single assay, clinical cancer gene test may be appropriately used for clinical use.

In this study, we aimed to perform CGP on tumor specimens from patients with lung adenocarcinomas who tested negative for *EGFR*, *KRAS* and *ALK* previously at our institution. Our ultimate goal was to identify patients who were candidates for targeted therapy and expand treatment choices for apparently “wild type” patients.

## RESULTS

### Patients

Clinical characteristics of patients are shown in Table 1. This study included 51 patients (18 males and 33 females) with a median age of 58 years (range: 29-77 years). All patients had adenocarcinoma histology and the majority of patients were never-smokers (76%, n=39). Twenty-five patients (49%) were stage 4 at the time of diagnosis. All patients had underwent *EGFR/KRAS/ALK* screening by conventional sequencing and FISH and were found to be triple-negative.

**Table 1:** Clinicopathologic findings of patients (N=51)

	N	%
**Gender**		
Male	18	35
Female	33	65
**Age**		
Median (range)	58 (29-77)	
**Pathology**		
Adenocarcinoma	51	100
**Stage**^a^		
I	5	10
II	8	16
III	13	25
IV	25	49
**Smoking**		
Never	39	76
Former or current smoker	12	24

a Clinical stage at the time of initial diagnosis was determined according to the 7^th^ American Joint Commission on Cancer guidelines

### Genomic alterations

Tumors were sequenced with a median coverage of 564x and a total of 190 known and 601 unknown individual GAs were identified. One or more GAs were uncovered by CGP from 94% (n= 48/51) of patients, with average of 3.7 alterations (range 0-10) per patient (Supplementary Table S1).

**Figure 1:**
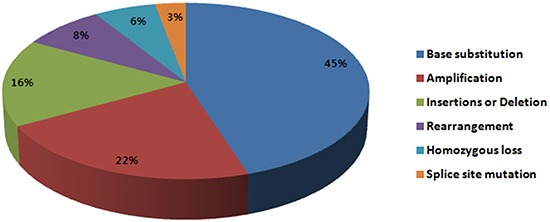
Frequency of genomic alterations identified in this study

Non-synonymous base substitutions comprised 50% (80/190) of the detected alterations (Figure 1): 15% (29/190) were insertions or deletion, and 3% (5/190) splice site mutation. *TP53* was the most commonly mutated gene (30%, n=24/80) among non-synonymous base substitutions, followed by *KRAS* (10%, n=8/80) and *EGFR* (10%, 8/80). Insertions or deletions commonly involved *TP53* (17%, 5/29) and *ERBB2* (14%, 4/29), and splice site mutations occurred in *TP53, INPP4B, ATR,* and *MAP2K4*. Gene amplification comprised 20% (39/190) of genomic alterations, and *MDM2* amplification was identified most frequently (13%, 5/39). Interestingly, *MDM2* amplification was frequently found with *CDK4* amplification in 3 out of 5 cases observed. Homozygous loss comprised 5% (10/190) of all genomic alterations and all 10 cases were observed with *CDKN2A*. Fusion genes were found in 7% (n=14/190) and most commonly involved *ROS1* fusion (50%, n=7/14). Alterations in the PI3K/mTOR pathway such as *PIK3CA* mutation, *AKT1* mutation, *PIK3R2* mutation, *STK11* inactivating mutation, *MTOR* mutation and *RICTOR* amplification were detected in 17 cases (33%).

Of note, *EGFR* (n=8) and *KRAS* (n=8) mutations, which were previously not detected by conventional sequencing, were identified and were mutually exclusive. *EGFR* mutations were enriched in tumors from females (*P* = 0.03). Of 8 *EGFR* mutations, 3 were not previously covered by the primer in the detection kit (M137I, Y891C, D770A). Of 8 *KRAS* mutations, 2 were not previously covered by the primer in the detection kit (Q61H, K179N). Mutations in tumor suppressor genes including *STK11* (n=4), *NF1* (n=2), *RB1* (n=2), *CDKN2A* (n=4) were observed. Mutations in chromatin modifying genes *ARID1A* (n=2), *MLL2* (n=2) were also observed.

### Clinically relevant genomic alterations

Based on National Comprehensive Cancer Network (NCCN) guidelines, we identified actionable genomic alterations with a matched targeted agent. Sixteen patients (39%) had previously known driver alterations: *BRAF* mutation [n=1], *EGFR* mutation [n=8], *ERBB2* mutation [n=4], *MET* amplification [n=1], *KIF5B-RET* rearrangement [n=2], *CCDC6-RET* rearrangement [n=1], *CD74-ROS1* rearrangement [n=1], *EZR-ROS1* rearrangement [n=5], and *SLC34A2-ROS1* rearrangement [n=1].

In addition, genomic alterations for which targeted therapy could be considered in clinical trials were discovered in 14 patients (27%). These include the following alterations and the corresponding therapy: *NF1* mutation (MEK inhibitor, NCT01885195), *KRAS* mutation (MEK inhibitor, NCT00890825) *CDKN2A* loss and *CDK4* amplification (CDK4/6 inhibitor, NCT01237236), *MDM2* amplification (MDM2 inhibitor, NCT01877382) and *PIK3CA* mutation (PI3K inhibitor, NCT01570296).

**Figure 2:**
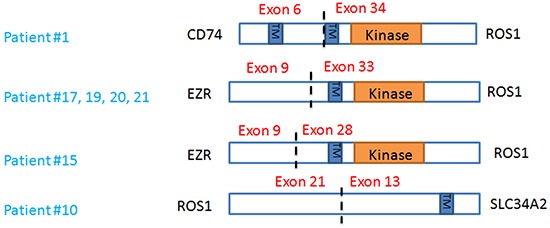
Characterization of *ROS1* rearrangements

Seven patients with *ROS1* rearrangements were enrolled in an ongoing trial (NCT01964157) and received ceritinib, a ROS1 inhibitor. *CD74-ROS1, EZR-ROS1, SLC34A2-ROS1* rearrangements were identified and detailed characterization of ROS1 rearrangements are outlined in Figure 2. Of note, one patient had *ROS1* gene without kinase domain fused with *SLC34A2*.

## DISCUSSION

In this study, we found that 16 of 51 (31%) patients harbored clinically actionable alterations that were not previously discovered in the routine clinical testing. This finding indicates an important opportunity to use targeted therapeutic strategies which will affect overall survival of these patients. In fact, 7 patients with *ROS1* rearrangement received ceritinib and 6 patients showed objective radiologic responses.

In recent years, the treatment of lung adenocarcinoma has been advanced by the development of multiple targeted therapies against receptor tyrosine kinases, RAS and RAF pathways. Still, lung adenocarcinomas without known oncogenes are treated with platinum-double chemotherapy as the first-line treatment. It is well-known that platinum-based chemotherapy has generated a plateau in the overall response rate (25-35%), time to progression (4-6 months) and the median overall survival (8-10 months). Our analysis of examining oncogene-negative lung adenocarcinomas expands the range of possible targetable alterations that may be applied to patients who have no other options but chemotherapy. For example, there were 16 patients with previously known driver alterations, and vemurafenib, crizotinib, afatinib, cabozantinib could be used for *BRAF* V600E mutation, *MET* amplification, *ROS1* rearrangements, *HER2* mutations and *RET* rearrangements, respectively.

A similar study was recently published which identified actionable GAs in lung adenocarcinomas using broad, hybrid, capture-bsed NGS [7]. In this study, NGS identified actionable GAs in 65% of tumors from never or light smokers deemed wild type by extensive non-NGS testing. Never or light smokers (≤15 pack years) were selected to enrich the diagnostic yield for potential drivers, but this approach may miss important driver oncogenes such as BRAF and KRAS mutations which are enriched in tumors from patients with a history of smoking. A recent study of using molecularly matched therapy in lung cancer has shown that patients whose genomic alterations were matched to a targeted therapy lived significantly longer than those who received non-targeted therapies [8]. In this study, Kris *et al.* identified a driver oncogene in 64% of tumors in which no such genomic alteration was initially found on non-NGS testing. Discordant results between the conventional sequencing and CGP imply that potentially clinically actionable targets may not be detected from in clinical care. Similarly, *EGFR* and *KRAS* mutations were uncovered in 16 (31%) patients in our study. Possible reasons for the discordant results are low sensitivity of detection method and intratumoral heterogeneity. Genomic alterations with low allele frequencies lead to false-negative results on conventional sequencing [9], and subclonal mutations may be heterogeneous according to biopsy sites [10].

Of note, alterations potentially predicting sensitivity to cyclin-dependent kinase (CDK) inhibitors such as *CDKN2A* loss, *CDK4* amplification, *CCND1* amplification were common in our patients, with 21 cases in total [11, 12]. Among these, the most frequently altered gene was *CDKN2A* homozygous loss, with 10 events. *MCL1* amplifications, which preclinical studies have shown to be associated with sensitivity to CDK inhibitors, were also found in 2 patients [13]. Co-amplification of *CDK4* and *MDM2* were noted in 3 patients. *CDK4* is located on the band q13 of chromosome 12, and this region contains *MDM2*, which is often amplified together in a variety of sarcomas such as liposarcoma, and rhabdomyosarcoma [14]. It is not yet known whether co-amplification of *CDK4* and *MDM2* are predictive biomarkers of CDK inhibitors, and functional validation in laboratory may be warranted in the future.

Genomic alterations that affect the PI3K-AKT-mTOR pathway were also frequent, with 17 cases (33%) identified. Although functional consequences of these alterations are not all clear, one patient harbored a *PIK3CA* E542K mutation, which is previously reported to activate the PI3K-AKT pathway and could benefit from the use of PI3K inhibitor [15].

Although TCGA group has identified *NF1* to be substantially mutated in adenocarcinomas [5], there was none identified in our patients. Since the majority of patients were never-smokers, it is reasonable that *NF1* mutations are differentially enriched in transversion-high tumors. In addition, *MET* activation through exon 14 skipping has recently been identified as unique molecular subtype of NSCLC [16]. In this study, we identified 1 patient who harbored *MET* mutation (S163Y) with concurrent *MET* genomic amplification. It is questionable that this patient may show response to c-MET inhibitor such as crizotinib.

In conclusion, we note that more comprehensive genomic characterization of the tumor reveals actionable alterations in triple-negative lung adenocarcinoma patients. All patients who received matched targeted therapy derived clinical benefit showing objective responses or evidence of tumor shrinkage. This study highlights previously unappreciated genetic alterations, enabling further refinement in sub-classification for the improved personalization of lung cancer treatment.

## MATERIALS AND METHODS

### Patient selection

Patients with lung adenocarcinomas who visited Yonsei Cancer Center (Seoul, Republic of Korea) between 2013 and 2015 were identified. Eligible patients had previously underwent standard molecular testing for *EGFR*/*KRAS* mutation and *EML4-ALK* rearrangement, and were tested triple-negative. These patients were enrolled in the prescreening part of an ongoing phase 2 trial, “An open-label, multicenter, phase 2 study of LDK378 in patients with non-small cell lung cancer harboring *ROS1*-rearrangement” (NCT01964157). Tissue and clinical information of patients were collected under a protocol approved by the Institutional Review Board of Severance Hospital. All patients provided written informed consent for the genetic analysis.

### Conventional testing for *EGFR*, *KRAS* and *EML4-ALK*

*EGFR* and *KRAS* mutations were determined by PNAClamp mutation detection kits (Panagene Inc., Daejeon, Republic of Korea). A total of 40 known mutations are detected in the exons 18-21 of *EGFR* gene, and a total of 14 mutations are detected in the exons 2-3 of *KRAS* gene. Break-apart fluorescent *in situ* hybridization (FISH) assays (Abbott Molecular, Inc. USA) were used to screen for *EML4-ALK* rearrangement, and positivity was defined as ≥ 15% of break-apart signals as described previously [17].

### NGS-based comprehensive genomic profiling assay

The archived tissue samples were histologically reviewed by an experienced pathologist (H.S.S) and analyzed by CGP at Foundation Medicine. Submitted specimens underwent additional pathologic review to make sure tissue adequacy (≥ 20% tumor nuclei and ≥ 50ng of DNA) before testing. DNA was extracted from FFPE samples and quantified by a Picogreen fluorescence assay. After DNA extraction of 50-100ng, library construction and hybrid capture of 4,557 exons of 287 cancer-related genes and 47 introns of 19 genes frequently rearranged in cancer was performed. Hybrid capture libraries were sequenced to > 500x average coverage with >100x at >99% of exons using Illumina HiSeq 2500 sequencer. A customized analysis pipeline was used to process sequencing data and genomic alterations such as base substitutions, short insertions and deletions, copy number alterations and genomic rearrangements were detected and reported [6].

### Data collection

Medical records of all patients and radiologic images were reviewed to evaluate demographic and clinicopathologic parameters, tumor response and survival outcome using a predesigned data collection format. Never-smokers were defined as those with a lifetime smoking-dose less than 100 cigarettes. Tumor response was assessed by a computed tomography scan in accordance with the Response Evaluation Criteria in Solid Tumors (RECIST) version 1.1 [18].

## SUPPLEMENTARY TABLES




