# Supplementary material for: Genomic profiling of lung adenocarcinoma patients reveals therapeutic targets and confers clinical benefit when standard molecular testing is negative
Source: Oncotarget. 2016 Mar 16;7(17):24172–8. doi: 10.18632/oncotarget.8138 (PMC5029692; doi:10.18632/oncotarget.8138)
Supplement: Supplementary file 1 [file oncotarget-07-24172-s001.pdf]

SUPPLEMENTARY TABLE

Supplementary Table S1: List of all genetic alterations identified in this study

See Supplementary File 1
